# How Viscoelastic Effects Impact Polymer Fluid Flow in Porous Media

**DOI:** 10.1007/s11242-026-02312-6

**Published:** 2026-05-14

**Authors:** Yongxin Wang, Si Suo, Callum Cuttle, Christopher W. MacMinn, Martin J. Blunt, Catherine O’Sullivan

**Affiliations:** 1 Department of Civil and Environmental Engineering, Imperial College London, London, SW7 2AZ UK; 2 Department of Earth Science and Engineering, Imperial College London, London, SW7 2AZ UK; 3 Department of Engineering Science, University of Oxford, Oxford, OX1 3PJ UK

**Keywords:** Viscoelasticity, Polymeric fluids, Porous media, Shear thinning rheology, 0000, 1111

## Abstract

**Abstract:**

Polymer fluids are widely used in subsurface and geotechnical engineering applications. While the steady shear rheology of polymer fluids is known to be reasonably captured by a Carreau-like shear-thinning model, it is still not fully understood how their elastic rheological characteristics, beyond shear-thinning behavior alone, influence their flow in porous media. In this study, we numerically investigate these effects using direct, pore-scale numerical simulations. By comparing data from simulations using the FENE-P model, which incorporates viscoelastic effects, with data from corresponding simulations using the Carreau model, which captures only shear thinning, we confirm that fluid elasticity can induce recirculation upstream of restrictions, leading to a reduction in polymer fluid conductance in porous media. As this recirculation is controlled by the geometric conditions, we conducted detailed comparisons between a two-dimensional model, a three-dimensional model mimicking microfluidics experiments, and an axisymmetric model, analogous to a constricted capillary tube. We also simulate flow in an ordered packing of uniform spheres to develop an understanding of the implications for flow in a 3D porous material. We find that these flows are regulated by the interplay between shear-thinning and elasticity effects. When the shear-thinning effect is sufficiently strong, the effects of elasticity are suppressed. In subsurface applications, viscoelastic effects are significant due to pore-scale confinement and fluid rheology itself, requiring explicit consideration in modeling, pilot design, and performance forecasting.

**Article Highlights:**

- In porous media, the pore geometries control the flow patterns of polymer fluids that have both shear-thinning and elastic characteristics.
- Upstream recirculation in a single-constriction channel induced by elastic forces takes place in wider pore spaces and develops with increasing Weissenberg number.
- For flow through a quasi-3D single constriction and a regular lattice packing, a greater energy loss is observed in the case of viscoelastic fluids in comparison with that in the corresponding Carreau fluid.
- The ratio of the zero-shear-rate viscosity to the infinite-shear-rate viscosity changes the polymer fluid conductance and flow patterns in a single-constriction channel.
- The extent of the upstream recirculation is proportional to the excessive pressure gradient in the viscoelastic fluid over the corresponding equivalent Carreau fluid.

**Graphical abstract:**

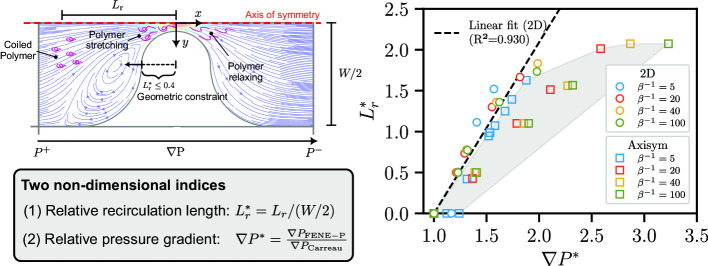

## Introduction

Polymer fluids are materials composed of long-chain macromolecules that are either dispersed or dissolved in a solvent, or exist in a molten state. These fluids can span a wide range of polymer concentrations, from dilute and semidilute solutions to concentrated solutions and pure polymer melts. Here, we focus specifically on semi-dilute aqueous solutions of long-chain polymers, such as partially hydrolyzed polyacrylamides (HPAM), which are commonly employed as viscoelastic fluids in studies of shear and extensional rheology, flow instabilities, and microfluidic flows. Below, we use the term “polymer fluids” to refer specifically to these solutions. The flow of polymer fluids in porous media underpins a wide range of engineering and industrial applications, including improved oil recovery (Sorbie 2013), remediation of groundwater contamination (Sandiford 1964; Smith et al. 2008; Wei et al. 2014), polymer extrusion, food processing, biological flows (Dullien 2012), and excavation support in geotechnical engineering (Lam et al. 2015a; Lam and Jefferis 2017; Ejezie et al. 2021). However, polymer fluids are well known to have a complex, viscoelastic, and shear-thinning rheology (Chen et al. 2010). They exhibit viscoelasticity as a result of the flow-driven deformation of the polymer chains. Their flow behavior becomes even more complex in porous media, as the surrounding solid matrix significantly influences spatial and temporal flow characteristics (Zami-Pierre et al. 2016; Kawale et al. 2017; Blunt 2017).

Stretching and releasing polymer chains can change the flow pattern, leading to flow recirculation upstream of obstacles in porous media, which adds to the energy dissipation of the flow (Evans and Walters 1986; Boger 1987; Purnode and Crochet 1996; Rodd et al. 2005). An enhanced pressure drop has been observed in some prior experimental data. In studies by Rothstein and McKinley (1999, 2001), pressure drops exceeding those of the reference Newtonian fluid were observed in a single constriction. This increased pressure drop was associated with the onset of vortex formation upstream. The complex morphology of most porous media leads to both shear and extensional flows, resulting in elongation of the polymer chains. Subsequent microfluidic experimental work has shown that the nonlinear state possesses the characteristics of elastic turbulence (Rodd et al. 2005; Kawale et al. 2017; Qin et al. 2019a; Browne et al. 2020; Browne 2022), and a nonlinear relationship between pressure drop and flow rate (Varshney and Steinberg 2017; Qin et al. 2019a).

Generalized Newtonian fluid models, such as the power-law model and the Carreau model, do not include viscoelasticity (Qin et al. 2019b; Browne et al. 2020; Kumar et al. 2021; Ekanem et al. 2020). However, the FENE-P (Finitely Extensible Nonlinear Elastic-Peterlin) rheological model (Bird et al. 1987) can be used to capture both the shear-thinning effect and viscoelasticity, explicitly incorporating polymer elasticity and the finite extensibility of the chains. The FENE-P model is well established. Previous simulation-based investigations have used the FENE-P rheological model to study the flow of a viscoelastic fluid past an array of cylindrical obstacles (Varchanis et al. 2020; Peng et al. 2023) and more complex porous media geometries (De et al. 2017; Kumar et al. 2021; Chen et al. 2024). However, the majority of numerical studies have been limited to 2D geometries, which fail to capture the true flow behavior of viscoelastic fluids in realistic porous media.

Several studies have also focused on comparing viscoelastic fluids with reference Newtonian fluids and inelastic shear-thinning fluids (Rodd et al. 2005; Pérez-Salas et al. 2024; Raihan et al. 2025). Rodd et al. (2005) conducted experiments in the planar contraction–expansion channel for different fluid rheologies: PEO and water. Different responses were observed in which PEO generated upstream vortices, while downstream vortices were induced by water; the former were attributed to elastic effects, whereas the latter were attributed to inertia. Pérez-Salas et al. (2024) investigated several rheological models that share the same shear-thinning behavior in flow through a 4:1 planar contraction. Their findings indicated that pressure drop is strongly governed by the shear-thinning characteristic, which is noticeably affected by elasticity. However, there is still a poor understanding of the effects of geometry and shear-thinning strength on the polymer fluid conductance, especially in realistic 3D porous media.

The objectives of this study are: (1) to explore the effects of geometry on the onset of fluid recirculation at low Reynolds number; (2) to investigate the conductance of polymer fluids in porous media, i.e., the relative pressure gradient, particularly when viscoelastic effects are pronounced; (3) to investigate how the degree of shear-thinning influences flow characteristics and the conductance of polymer fluids; and (4) to quantify the induced upstream flow recirculation and link it to the relative pressure gradient. To achieve these objectives, we adopt the Carreau model (Carreau 1972), which captures the shear-thinning rheology only, and the FENE-P model (Bird et al. 1980), which captures both shear-thinning effects and viscoelasticity. We used direct, pore-scale numerical simulations to study flow in single-constriction channels with different geometric configurations, followed by a regular lattice packing to be representative of a realistic pore system.

## Methodology

### Governing Equations and Numerical Method

For flow of an incompressible fluid, conservation of mass and linear momentum can be written as

1 $$\begin{aligned} &  \nabla \cdot {\textbf {u}}=0 \end{aligned}$$

2 $$\begin{aligned} &  \rho \left( \frac{\partial {\textbf {u}}}{\partial t} + {\textbf {u}}\cdot \nabla {\textbf {u}}\right) =-\nabla p+\nabla \cdot \boldsymbol{\tau } \end{aligned}$$

where $\rho$, ${\textbf {u}}$, and *p* are fluid density, velocity, and pressure, respectively. For a shear-thinning but inelastic fluid (e.g., a Carreau fluid), the deviatoric viscous stress tensor $\boldsymbol{\tau }$ is expressed by

3 $$\begin{aligned} \boldsymbol{\tau }=2\mu (\dot{\gamma }){\textbf {D}} \end{aligned}$$

where ${\textbf {D}}=\frac{1}{2}\left[ \nabla \boldsymbol{\textrm{u}}+(\nabla \boldsymbol{\textrm{u}})^{\textrm{T}}\right]$ is the rate-of-strain tensor, $\dot{\gamma }=\sqrt{2{\textbf {D}}:{\textbf {D}}}$ is the scalar shear rate, and $\mu$ is the dynamic viscosity, as defined, for example, by the Carreau model (Eq. (4)). For a viscoelastic fluid, the viscoelastic stress tensor $\boldsymbol{\tau }$ (i.e., the “extra” stress tensor) is no longer purely deviatoric and can be divided into a solvent contribution ($\boldsymbol{\tau }_\textrm{s}$) and a polymeric contribution ($\boldsymbol{\tau }_\textrm{p}$), $\boldsymbol{\tau }=\boldsymbol{\tau }_\textrm{p}+\boldsymbol{\tau }_\textrm{s}$. The solvent stress tensor, $\boldsymbol{\tau }_\textrm{s}$, can be obtained by $\boldsymbol{\tau }_\textrm{s}=\mu _\textrm{s}\left[ \nabla \boldsymbol{\textrm{u}}+(\nabla \boldsymbol{\textrm{u}})^{\textrm{T}}\right]$ and the polymeric stress $\boldsymbol{\tau }_\textrm{p}$ is specified by, for example, the FENE-P constitutive equation (presented below).

All numerical simulations using the FENE-P rheological model were performed using the rheoFoam solver of the open-source code OpenFOAM^®^ integrated with RheoTool (Pimenta and Alves 2016). The spatial gradient and divergence operators were discretized with the Gauss linear scheme and temporal derivatives with the Euler scheme. Advective terms in the momentum and constitutive equations were treated using the high-resolution CUBISTA scheme (Alves et al. 2003). The stress field was solved with a preconditioned biconjugate gradient solver (PBiCG) and a DILU preconditioner (Lee et al. 2003), with a relative tolerance of $10^{-10}$. The velocity and pressure fields were solved using the preconditioned conjugate gradient (PCG) solver and a diagonal-based incomplete Cholesky (DIC) preconditioner, also with a relative tolerance of $10^{-10}$. The SIMPLE method was applied to decouple velocity and pressure. More details on pressure–velocity and stress–velocity coupling and the overall solution procedure can be found in Pimenta and Alves (2016). Convergence at each time step was assessed by monitoring the residuals of all governing equations, which were reduced below a relative tolerance of $10^{-6}$ for the stress, velocity, and pressure fields before advancing in time.

### Rheological Models

#### Carreau Model

The Carreau model (Carreau 1972; Carreau et al. 1979) is a generalized Newtonian model (viscous and inelastic) that has been widely adopted to describe shear-thinning rheology. The model includes three flow regimes: the upper Newtonian plateau at low shear rates ($\mu \rightarrow \mu _0$ for $\dot{\gamma }\ll \lambda ^{-1}$), a power-law decay at intermediate shear rates ($\mu \propto \dot{\gamma }^{(n-1)}$), and the lower Newtonian plateau at high shear rates ($\mu \rightarrow \mu _\infty$ for $\dot{\gamma }\gg \lambda ^{-1}$), where $\lambda ^{-1}$ is a constant material property. The Carreau model can be expressed as

4 $$\begin{aligned} \frac{\mu -\mu _\infty }{\mu _0-\mu _\infty }=\left[ 1+(\lambda \dot{\gamma })^2\right] ^{-(1-n)/2} \end{aligned}$$

where $n\le 1$ determines the power-law exponent.

#### FENE-P Model

The FENE-P model (Finitely Extensible Nonlinear Elastic-Peterlin) (Bird et al. 1980) is a classical viscoelastic rheological model, capturing both elastic deformation and viscous flow (De et al. 2017; Aramideh et al. 2019; Kumar et al. 2021; Buza et al. 2022; Kumar et al. 2023b). Conceptually, the FENE-P model represents polymer chains as elastic “dumbells” consisting of two beads connected by a finitely extensible, nonlinear spring that convects and aligns with the flow. As the shear rate increases, polymers in the FENE-P model become increasingly stretched and aligned until they near their finite maximum extension. Consequently, the polymer stress grows more slowly than the shear rate, causing the apparent viscosity to decrease with increasing shear and thus exhibiting shear-thinning behavior. The FENE-P model is given in the following stress tensor:

5 $$\begin{aligned} \boldsymbol{\tau _\textrm{p}}+\lambda _\textrm{F}\left( \frac{\overset{\nabla }{\boldsymbol{\tau _\textrm{p}}}}{f(\boldsymbol{\tau _\textrm{p}})}\right) =2a\mu _\textrm{p} {\textbf {D}}\left( \frac{1}{f(\boldsymbol{\tau _\textrm{p}})}\right) -a\mu _\textrm{p} {\textbf {I}}\frac{\textrm{D}}{\textrm{D}t}\left( \frac{1}{f(\boldsymbol{\tau _\textrm{p}})}\right) \end{aligned}$$

6 $$\begin{aligned} f=\frac{L^2+\frac{\lambda _\textrm{F}}{a\mu _\textrm{p}}\textrm{tr}(\boldsymbol{\tau _\textrm{p}})}{L^2-3},~\text {with}~a=\frac{L^2}{L^2-3} \end{aligned}$$

where $\lambda _\textrm{F}$ is the relaxation time of the polymer, *a* is a material constant, $\mu _\textrm{p}$ is the polymeric contribution to the zero-shear-rate viscosity, ${\textbf {I}}$ is the identity tensor, and $\frac{D}{Dt}$ is the material time derivative following the fluid. $L^2=3R_0^2/R_e^2$ measures the extensibility of polymer chains (Purnode and Crochet 1998; Bird et al. 1980) with $R_0$ being the maximum chain length and $R_e$ the equilibrium chain length (Bird et al. 1987; Sorbie 2013). Typical values of $L^2$ are in the range $10\sim 1000$ (Aramideh et al. 2019; Kumar 2022). In the limit $L^2\rightarrow \infty$, the FENE-P model reduces to the Oldroyd-B model (Oldroyd 1950), which does not exhibit shear thinning. Large values of $L^2$ may induce a pronounced fluctuating flow (sometimes called elastic turbulence) with variation in time. In this study, a low value of $L^2$ ($=10$) was used to achieve a steady flow without fluctuations. More details are found in Appendix A.

#### Model Parameters

**Fig. 1:**
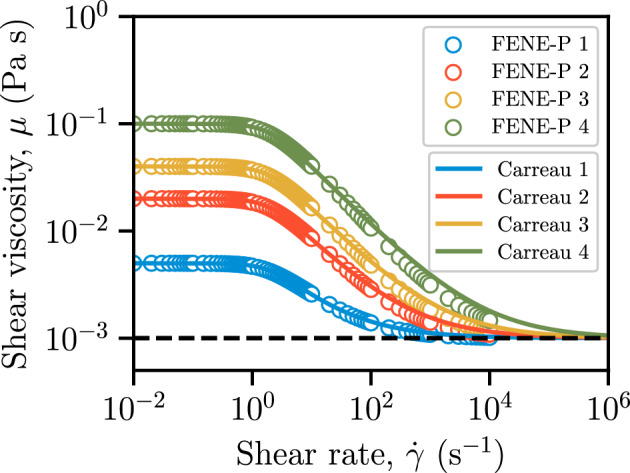
Steady, homogeneous shear rheology for four sets of FENE-P parameters (symbols), each with corresponding Carreau fit (curves). The solvent viscosity, $\mu _\textrm{s}$, is fixed as $1~\mathrm {mPa\cdot s}$ (i.e., the viscosity of water; dashed horizontal line). Parameters are given in Tables 1 and 2

Computational considerations limited the range of FENE-P model parameters that could be adopted in the simulations. Employing small values of $L^2$ confines this study to polymer solutions with short polymer chains (smaller $L^2$ means weaker elasticity), which are insufficient to induce time-dependent behavior. To enable a direct comparison between a Carreau fluid and a FENE-P fluid, we simulate the steady shear rheology for each set of FENE-P parameters and then fit a Carreau model to the result. Specifically, Table 1 lists four sets of FENE-P model parameters with four values of the viscosity ratio $\beta ~(=\frac{\mu _\textrm{s}}{\mu _\textrm{s}+\mu _\textrm{p}})$ to characterize the shear-thinning strength ($\beta ^{-1}$) of the fluid and a fixed value of $L^2=10$ to achieve numerical stability. A low value of $\beta ^{-1}$ signifies a fluid whose viscosity remains nearly constant. The solvent viscosity, $\mu _\textrm{s}$, is fixed as $1~\mathrm {mPa\cdot s}$ (i.e., the viscosity of water). We take the fluid density $\rho$ and the relaxation time $\lambda _\textrm{F}$ to be constant at $1000~\mathrm {kg/m^3}$ and $1.0~\textrm{s}$, respectively. The rheological curves for these FENE-P model parameters were obtained using OpenFOAM^®^ integrated with RheoTool (Pimenta and Alves 2016). A uniform shear rate $\dot{\gamma }$ was produced in a single hexahedral computational cell ($1\times 1\times 1$) to mimic a rheometric test under the same experimental conditions. The Carreau model parameters (Carreau 1972) that gave an optimal fit to the FENE-P rheological curves after fixing the upper and lower Newtonian plateaus are presented in Table 2. Figure 1 presents the variation in shear viscosity with the shear strain rate under steady, homogeneous, planar shear for four sets of FENE-P parameters and their corresponding Carreau fits.

The fluid rheology adopted here does not correspond directly to the properties of polymer support fluids due to the constraints of the FENE-P constitutive model and computational limitations. For example, the shear-thinning strength of polymer support fluids used in ground engineering applications can reach $\beta ^{-1}=10^5$ (e.g., Lam et al. (2025b)). However, the fluid conductance and patterns that we observe can be mapped to real-world conditions by matching the appropriate non-dimensional groups, performing inverse parameter identification and validating within the relevant flow regimes, as discussed in Sect. 3.2.

**Table 1:** Values of different FENE-P parameters used in this study (see Eq. 5). The shear-thinning strength parameter ($\beta ^{-1}$) is listed in the last column

Name	$\rho ~(\mathrm {kg/m^3})$	$\lambda _\textrm{F}~(\textrm{s})$	$\mu _\textrm{s}~(\text {mPa s})$	$\mu _\textrm{p}~(\text {mPa s})$	$L^2~(-)$	$\beta ^{-1}~(-)$
FENE-P 1	1000	1	1	4	10	5
FENE-P 2	1000	1	1	19	10	20
FENE-P 3	1000	1	1	39	10	40
FENE-P 4	1000	1	1	99	10	100

**Table 2:** Fitted Carreau model parameters associated with the simulated steady, homogeneous shear rheology of each of the four sets of FENE-P parameters in Table 1 (see Eq. 4)

Name	$\mu _0~(\text {mPa s})$	$\mu _\infty ~(\text {mPa s})$	$\lambda ~(\text {s})$	$n~(-)$
Carreau 1	5	1	0.5256	0.4371
Carreau 2	20	1	0.5256	0.4371
Carreau 3	40	1	0.5256	0.4371
Carreau 4	100	1	0.5256	0.4371

**Fig. 2:**
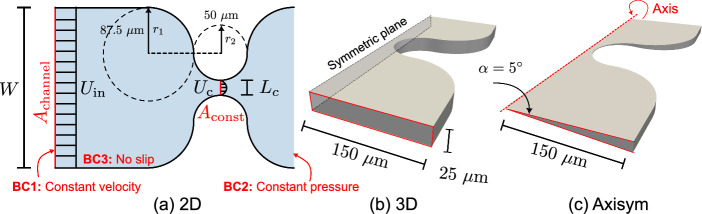
Detailed geometric dimensions in different single-constriction configurations: **a** 2D; **b** 3D; and **c** axisymmetric. The common boundary conditions are illustrated in (**a**). Empty boundaries are imposed on top and bottom walls for the 2D case, no-slip for the 3D case, and wedge boundary for the axisymmetric case. The choice of the constriction configuration is inspired by a microfluidic tests (Cuttle et al. 2025)

### Simulation Setup

In this study, we first examine viscoelastic flow through a single-constriction channel with varying pore geometries (2D, 3D, and axisymmetric models; see Fig. 2a–c). The channel has a constriction size of $L_\textrm{c}=25~\upmu$m and a channel width (or diameter) of $W=300~\upmu$m; the curvature of the constriction is illustrated in Fig. 2a. The shape of the constriction is inspired by a microfluidic test in Cuttle et al. (2025). The geometry resembles that used in previous studies featuring a sudden constriction (for example, Rodd et al. (2005)). Employing circular segments makes the geometry more directly comparable to other porous media models, such as spherical packings. A total channel length of $L=2000~\upmu$m was considered to allow for fully developed flow. The 2D case (Fig. 2a) can be regarded as planar, while in the case of the rectangular 3D case (Fig. 2b), the height (thickness) was taken as $H_c=25~\upmu$m (AR$=H_c/L_c=1$). This configuration is analogous to a single constriction in a microfluidic model of a porous material. A constricted pipe (a converging–diverging pipe) was adopted to further explore the effects of viscoelasticity on the flow pattern since it can better reveal the viscoelastic polymer fluid in a constriction in a more realistic 3D material. A computationally efficient axisymmetric case was used for these three-dimensional simulations, as illustrated in Fig. 2c. A constant and uniform inlet velocity, null value of the polymeric stress tensor ($\boldsymbol{\tau }_\textrm{p}$), and zero-gradient pressure were imposed as boundary conditions at the inlet of the capillary tube. At the walls, we imposed no-slip and no-penetration conditions for velocity, linear extrapolation for polymeric stresses, and zero normal gradient for pressure (Pimenta and Alves 2017). At the outlet, a pressure $p=0$ was prescribed, while zero normal gradients were imposed for the velocity components and the polymeric stress tensor ${\tau _\textrm{p}}$. In all cases, the simulation mesh was refined in the constriction region to ensure adequate accuracy and numerical stability. For simplicity, the terms *2D*, *3D* and *Axisym* are used below to refer to the scenarios illustrated in Fig. 2a–c, respectively.

**Fig. 3:**
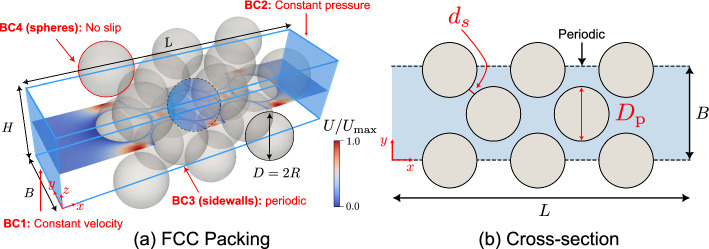
**a** Regular lattice packing (face-centered cubic) with periodic boundaries parallel to the flow direction. The colorbar represents the normalized velocity. **b** Plane view of the FCC packing. A constant distance of $2~\textrm{mm}$ (known as buffer layer) is imposed between the inlet (outlet) and the first (last) row of spheres for all regular lattice packings to achieve a fully developed flow

To explore the impact on a system of connected pores, we also consider a fully 3D, face-centered cubic (FCC) packing of uniform spheres (23 particles in this geometric configuration), using periodic boundaries to mimic a system of infinite lateral extent (Fig. 3a). The spheres have a radius of $R=0.495~\textrm{mm}$, and the domain has lateral dimensions (orthogonal to flow) of $B=H\approx 1.5~\textrm{mm}$ and a length *L* approx $7~\textrm{mm}$. This regular lattice configuration can be validated against the results of Zick and Homsy (1982). This small domain size facilitated the use of a very fine mesh (the ratio between the mesh size and the radius of the sphere particle, $R/\Delta d \approx 45$), enhancing the accuracy of computing the fluid–particle interaction forces. SnappyHexMesh, an OpenFOAM^®^ mesh generation algorithm, was used to create a grid of hexahedral cells (Greenshields 2021). Local refinement was applied to resolve the narrow channels between the spheres. The final mesh has around 1.6 million cells. Our previous study (Wang et al. 2025) included a study of mesh sensitivity for simulations using water, which informed the choice of mesh topology adopted here. The permeability is $K=4.8\times 10^{-10}~\mathrm {m^2}$ (Ejezie et al. 2021; Wang et al. 2025). Porosity could be systematically controlled with a constant fabric when using these regular packings. More details of this FCC simulation can be found in Wang et al. (2025). Similar boundaries were imposed at the inlet and outlet as in the case of single-constriction channels (see Sect. 3). The lateral boundary conditions were periodic for velocity, linear extrapolation for polymeric stresses, and zero normal gradient for pressure (Pimenta and Alves 2017). Our previous study showed how this configuration can be used to explain the link between viscosity and fluid–particle interaction forces when Carreau fluids permeate a granular material (Wang et al. 2025).

The Reynolds number ($\textrm{Re}$) and the Weissenberg number ($\textrm{Wi}$) are the relevant dimensionless numbers to characterize the flow for the simulations discussed here. The Reynolds number $\textrm{Re}=\rho U_cL/\mu _0$ represents the ratio of inertial to viscous stresses, where $\mu _0$ is the zero-shear-rate viscosity, $\rho$ is the fluid density, $U_c$ is the interstitial velocity, and *L* is the characteristic length. In a single constriction, *L* is taken as the constriction width at the narrowest point $L_c$ (see Fig. 2a); in a regular lattice packing, *L* is defined as the distance between adjacent particles $d_s$ (defined in Fig. 3b). We maintain $\textrm{Re}\ll 1$ across all of the scenarios studied here, so that inertia is negligible and the flow is always in the creeping-flow regime. The amount of viscoelasticity is characterized by the Weissenberg number (Dealy 2010), $\textrm{Wi}=\lambda _\textrm{F} U_c/L$, where $\lambda _\textrm{F}$ is the fluid relaxation time (defined in Eq. (5)), and $U_c$ is the interstitial velocity. In the single-constriction simulations (2D, 3D and axisymmetric), we take $\textrm{Wi}=\lambda _\textrm{F}\left( U_\textrm{in}\frac{A_\textrm{channel}}{A_\textrm{const}}\right) /L_c=\lambda _\textrm{F} U_c/L_c$, where $U_\textrm{in}$ is the inlet flow velocity, $A_\textrm{channel}$ and $A_\textrm{const}$ are the cross-sectional areas of the channel and the constricted throat, as illustrated in Fig. 2a. For the configuration of packed spheres, we take $\textrm{Wi}=\lambda _\textrm{F}\left( \frac{U_\textrm{in}}{\phi }\right) /d_s$, where $\phi$ is the porosity of the sphere packing ($\phi =0.35$ in this study). Here we varied the flow velocity $U_\textrm{in}$ to change $\textrm{Wi}$, while keeping $\lambda _\textrm{F}=1.0~\textrm{s}$, $L^2=10$, and $\rho =1000~\mathrm {kg/m^3}$. For different geometric configurations (varying cross-sectional areas upstream and downstream), the same value of $\textrm{Wi}$ can be achieved by keeping the same interstitial velocity $U_c$. This facilitates a detailed subsequent comparison of fluid rheology without changing the rheological curves as $\textrm{Wi}$ increases.

### Validation and Benchmarking

**Fig. 4:**
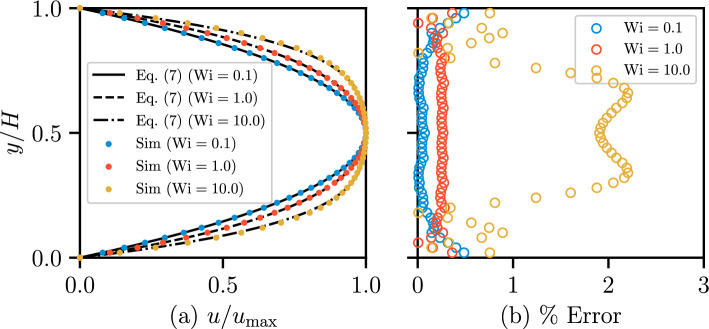
**a** Normalized velocity profile of FENE-P fluids in a 2D straight channel from numerical simulations (dots) and from the analytical solution (Eq. 7; curves) for three different values of $\textrm{Wi}$ ($\mu _\textrm{s}=0~\mathrm {Pa\cdot s}$, $\mu _\textrm{p}=0.004~\mathrm {Pa\cdot s}$, $\lambda _\textrm{F}=1.0~\textrm{s}$, and $L^2=10$). **b** Profile of relative error for three different values of $\textrm{Wi}$

To confirm the ability of the open-source code OpenFOAM^®^ integrated with RheoTool (Pimenta and Alves 2016) to simulate a simple shear flow using the viscoelastic FENE-P rheology, a 2D channel flow ($H=1~\textrm{mm}$ and $L=10~\textrm{mm}$) was first considered. Taking the solvent viscosity $\mu _\textrm{s}$ to be zero enables an analytical solution in a closed form for a FENE-P fluid, which is given by

7 $$\begin{aligned} \begin{aligned} u(y)&=A\nabla P(y^2-R^2)+B(\nabla P)^3(y^2-R^2)(y^2+R^2)\\&=\nabla P(y^2-R^2)\left[ A+B(\nabla P)^2(y^2+R^2)\right] \end{aligned} \end{aligned}$$

where

8 $$\begin{aligned} A=\frac{L^2+3a}{2a\mu _\textrm{p}L^2};\quad B=\frac{\lambda _\textrm{F}^2}{2a^2\mu _\textrm{p}^3L^2} \end{aligned}$$

and $\nabla P$ is the applied pressure gradient, *y* is the transverse distance from the center of the channel, $R=H/2$ is the half-width of the channel, and *a* is given in Eq. (6). Figure 4a compares the above analytical solution with the results of numerical simulations for three Weissenberg numbers, $\textrm{Wi}=\lambda _\textrm{F}U/H=0.1,~1.0,~\textrm{and}~10$. At $\textrm{Wi}=0.1$ and 1.0, the relative error in velocity is acceptably low (below 0.5%); at $\textrm{Wi}=10$, the maximum errors increase to approximately 2% as a result of the enhanced inertial effects arising from the shear-thinning rheology, as shown in Fig. 4b.

**Fig. 5:**
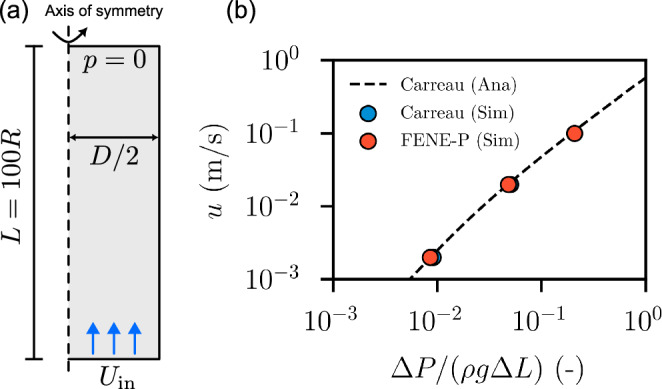
Benchmark of FENE-P viscoelastic simulations at $\textrm{Wi}=[1.4, 14, 71]$ (parameters for both FENE-P and Carreau fluids are listed in Tables 1 and 2). No distinct viscoelastic behavior is observed under simple, steady shear flow. The analytical solution (dashed line) for the Carreau model is implemented based on the work of Sochi (2015)

Next, we consider the flow of both FENE-P and Carreau fluids in a single 3D circular capillary tube $70~\textrm{mm}$ long and diameter $D=1.4~\textrm{mm}$ (model parameters are listed in Tables 1 and 2). The boundary conditions applied were identical to those used for the 3D single-constriction channel (see Sect. 2.3). An O-grid topology mesh was used with 3600 cells in total across the cross section (perpendicular to the direction of flow). The mesh was refined near the wall. A mesh sensitivity study (see Table 3) confirmed a negligible deviation between the data generated using the mesh adopted here (M1) and a finer mesh (M2 and M3). The effect of inertia is negligible in this case as $\textrm{Re}<0.1$, even at higher $\textrm{Wi}$ values. The Weissenberg number spans values between 1.4 and 71. In the case of a viscoelastic fluid, simple shear flows are expected in these cases. The relationship between the average velocity and head gradient is similar for Carreau and viscoelastic fluids, as illustrated in Fig. 5b. This is expected since the Carreau fluid was derived from the FENE-P fluid under steady, uniform shear. The results for both fluids closely correspond to the analytical solution for the Carreau fluid in a capillary tube, which is implemented based on Sochi (2015). The resulting data indicate that no clear viscoelastic effect is observed in a simple steady shear flow, as expected.

**Table 3:** Mesh sensitivity analysis of the pressure gradient in simple capillary tube at $u = 0.02~\mathrm {m/s}$ for Carreau model rheology “Carreau 1” (see Table 2) for three refinement levels

Mesh Scheme	No. of cells	Relative error (%)
M1	3600	2.79
M2	8000	1.97
M3	14400	0.97

The cell count refers to the cross-sectional plane normal to the flow direction. The relative error is defined as the difference between the simulation result and the analytical result, divided by the analytical value. The M1 meshing strategy is selected for the subsequent simulations, as it provides acceptably low errors

## Single Constriction

We now study a single constriction with 2D and quasi-3D geometries, as illustrated in Fig. 2a–c. A single set of FENE-P and equivalent Carreau model parameters was considered in the single-constriction simulations to investigate the geometric effects on the eddy formation and polymer fluid conductance. Next, four sets of parameters of the FENE-P model (Table 1) and their corresponding fitted Carreau curves (Table 2) were applied to investigate the effect of shear-thinning strength ($\beta ^{-1}$).

**Fig. 6:**
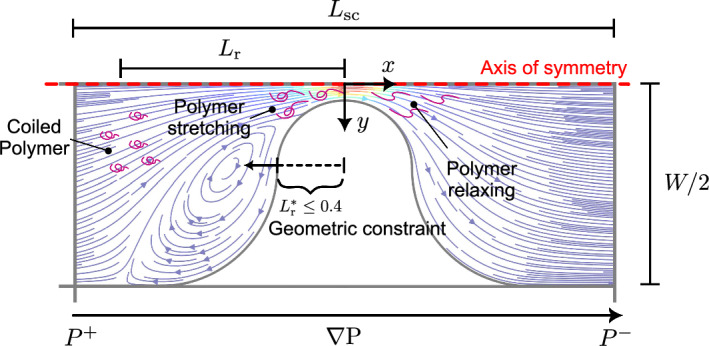
Determination of pressure gradient and recirculation length for three single-constriction cases. Pressure gradient is defined as $\nabla P=(P^+-P^-)/L_\textrm{sc}$. Note that the range $L_r^* = L_r/(W/2) \le 0.4$ corresponds to the constricted section, where no recirculation is expected due to the presence of the occupied constriction part

**Fig. 7:**
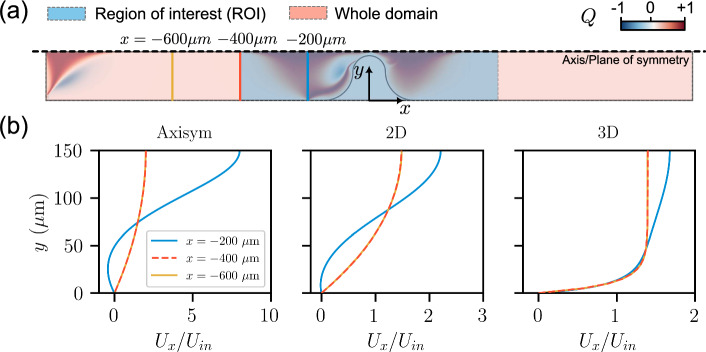
**a** Determination of the region of interest for calculating pressure drop for single-constriction channels. Slices at different *x* positions are marked with corresponding colors. The contour represents the distribution of flow topology parameter *Q* at $\textrm{Wi}=24$ for rheology “FENE-P 1”. **b** Velocity profiles at different *x* positions at $\textrm{Wi}=24$ in three geometric configurations. Fluid rheology of “FENE-P 1” was considered here (see Table 1). Note that red dashed line coincides with the yellow solid line. The velocities are not affected by the region distributed by the constriction in the selected slice (red), nor in the further upstream slice (yellow) with unchanged flow velocity profiles

To quantify the effects of elasticity on polymer fluid flow for the three geometries adopted, two non-dimensional indices are defined: the relative pressure gradient $\nabla P^*$ and the relative recirculation length $L_r^*$. The relative pressure gradient $\nabla P^*$ is defined as the pressure gradient in the FENE-P fluid relative to that in the Carreau fluid at the same flow velocity, $\nabla P^*=\nabla P_\mathrm {FENE-P}/\nabla P_\textrm{Carreau}$. In these single-constriction cases, the pressure gradient $\nabla P$ for Carreau and FENE-P fluids is calculated as $\nabla P=(P^+-P^-)/L_\textrm{sc}$, as illustrated in Fig. 6. $P^+$ and $P^-$ are the pressures along the centerline and at the same distance upstream ($x<0$) and downstream ($x>0$) from the constriction, respectively. $L_\textrm{sc}$ is the channel length considered for the calculation of the pressure drop, as demonstrated in Fig. 6. We take the length to be $L_\textrm{sc}=800 ~\upmu \textrm{m}$ (i.e., from $x=-400~\upmu \textrm{m}$ to $x=+400~\upmu \textrm{m}$), since the velocity profile is fully developed at these points in all three simulation configurations at $\textrm{Wi}=24$, as illustrated in Fig. 7a and b for rheology “FENE-P 1”. Note that “fully developed” means that velocities are not affected by the region distributed by the constriction with unchanged velocity profiles. In single-constriction cases, the recirculation length $L_r$ is defined as the distance between the most left of the recirculation (close to the channel wall where eddy separate from the main flow) and the center of the constriction ($x=0$), as illustrated in Fig. 6. We report our results below in terms of a non-dimensional recirculation length, $L_r^*=L_r/(W/2)$. Note that recirculation occurs only when $L_r^*>0.4$ due to the definition (see Fig. 6). For $L_r^*\le 0.4$, the solid region occupies the space of the fluid domain.

The flow topology parameter *Q* is used to characterize the geometric effects on the flow field (Perry and Chong 1987). The flow topology parameter *Q* is calculated as the second invariant of the velocity gradient tensor $\nabla \boldsymbol{\textrm{u}}$,

9 $$\begin{aligned} Q=\frac{\vert \boldsymbol{\textrm{D}}|-\vert \boldsymbol{\textrm{W}}|}{\vert \boldsymbol{\textrm{D}}|+\vert \boldsymbol{\textrm{W}}|} \end{aligned}$$

where $\vert \boldsymbol{\textrm{D}}|$ is the magnitude of the rate-of-strain tensor and $\vert \boldsymbol{\textrm{W}}|$ is the magnitude of the rate of rotation tensor ($\boldsymbol{\textrm{W}}=\frac{1}{2}\left( \nabla \boldsymbol{\textrm{u}}-(\nabla \boldsymbol{\textrm{u}})^\textrm{T}\right)$). *Q* has been used in some recent studies of non-Newtonian flows (Haward et al. 2016; Poole 2023) and allows three types of flow topology to be identified: pure rotational flow for $Q=-1~(\vert {\textbf {D}}|\equiv 0)$, simple shear flow for $Q=0~(\vert {\textbf {D}}|\equiv \vert {\textbf {W}}|)$, and pure elongational flow for $Q=+1~(\vert {\textbf {W}}|\equiv 0)$.

### Effects of Pore Geometry

#### Flow Patterns

**Fig. 8:**
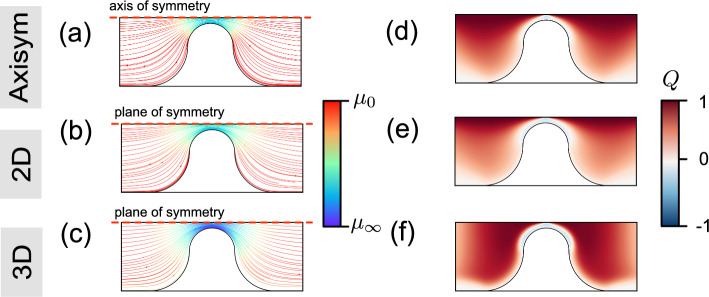
Flow profiles obtained for Carreau model rheology “Carreau 1” (see Table 2) at flow velocities corresponding to $\textrm{Wi}=24$ in the three different geometries. In the 3D case, the plane located at the mid-depth is shown. **a**–**c** Streamlines colored to represent the local shear viscosity for three different geometries. **d**–**f** spatial distribution of flow topology parameter *Q*. A more pronounced shear-thinning effect is observed in (**c**), the 3D case

The flow characteristics for the shear-thinning but inelastic Carreau model are first presented to give preliminary insights as to how the geometry affects flow. In the 3D case, the analysis focuses on the plane situated at the mid-depth. Figure 8a–c illustrates the streamlines and dynamic viscosity fields, respectively, for the three simulation configurations with rheology “Carreau 1” at the same interstitial flow velocity, $U_c=6\times 10^{-4}~\mathrm {m/s}$ (corresponding to $\textrm{Wi}=24$ in FENE-P fluids). In this example, flow recirculation does not occur for any of the three geometries. The spatial distribution of the dynamic viscosity in the axisymmetric and 2D cases behaves similarly, whereas the 3D case shows a more pronounced shear-thinning effect ($\mu \rightarrow \mu _\infty$). When polymer chains are strongly stretched and aligned by the flow, the effective local shear rate rises, which in turn decreases the local viscosity.

**Fig. 9:**
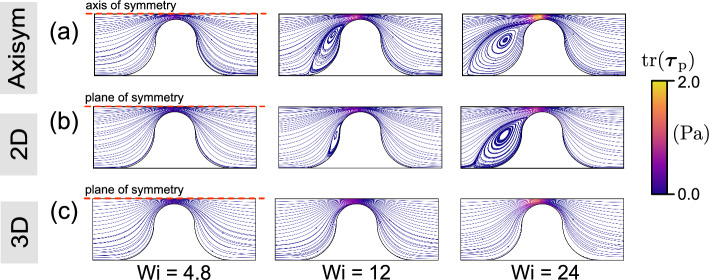
Streamlines colored by the magnitude of trace of the polymeric stress tensor in **a** an axisymmetric channel; **b** 2D channel; **c** 3D channel (mid-depth plane) at different values of $\textrm{Wi}$ for rheology “FENE-P 1”. $\textrm{Wi}=\lambda _\textrm{F}U_c/L_c$, where $U_c$ is the interstitial velocity, defined as $U_c=U_\textrm{in}(A_\textrm{channel}/A_\textrm{const})$. Differences in confinement change the size of the recirculation zone and the recirculation region evolves with $\textrm{Wi}$ as a result of larger polymer stretching and oriented by flow. No upstream recirculation was observed in the 3D channel, even at a high value of $\textrm{Wi}$

Turning to the viscoelastic case, Fig. 9 presents the streamlines colored by the value of the trace of the polymeric stress tensor ($\textrm{tr}(\boldsymbol{\tau }_\textrm{p})=\tau _{xx}+\tau _{yy}+\tau _{zz}$) for rheology “FENE-P 1” for the three $\textrm{Wi}$ values considered here. The quantity $\textrm{tr}(\boldsymbol{\tau }_\textrm{p})$ measures the elongation of the polymer chains (Kumar et al. 2021) and is considered as an indicator of elastic (effectively extensional) stress. Although this is not a direct measure of the classical extensional viscosity, a higher value of $\textrm{tr}(\boldsymbol{\tau _\textrm{p}})$ indicates a strong stretching of the polymer chains (strong elongational deformation), hindering fluid flow across high-stress areas and resulting in upstream recirculation (traditionally viewed as an elastic effect) even at low $\textrm{Re}$; the inertial response would lead to flow recirculation downstream.

At low $\textrm{Wi}$, upstream flow recirculations do not occur in any of the three geometries, as illustrated in the first column of Fig. 9. As shown in Fig. 9a and b, upstream recirculation appears and develops for the axisymmetric and 2D cases as $\textrm{Wi}$ increases. For this example, the polymeric stress overcomes the viscous stresses that tend to keep the flow laminar. Upstream of the constriction, streaks of large $\textrm{tr}(\boldsymbol{\tau }_\textrm{p})$ detach from the wall and migrate toward the centerline, suggesting that strong polymer elongation promotes the formation of recirculation zones (Kumar et al. 2023a). The onset of purely elastic instabilities is associated with the characteristic curvature of the flow and the stress along the streamlines, a relationship now recognized as the Pakdel–McKinley scaling (Pakdel and McKinley 1996; McKinley et al. 1996). Subsequent experimental and numerical studies in microchannel and obstacle flows show that most of these flows involve curved streamlines, and thus, their instability is attributed to hoop stress (e.g., elastic or polymeric stress)-driven instabilities (Browne et al. 2020; Datta et al. 2022). The low $\textrm{tr}(\boldsymbol{\tau }_\textrm{p})$ within the recirculation zones reveals that these zones reduce polymeric stress. Downstream of the throat, flow divergence allows polymer chains to relax, leading to an eddy-free downstream region. As depicted in Fig. 9, the polymeric stress $\textrm{tr}(\boldsymbol{\tau }_\textrm{p})$ increases with increasing $\textrm{Wi}$ values in all three geometries. At a given $\textrm{Wi}$, the axisymmetric configuration produces the highest polymeric stress, whereas the 2D configuration produces the lowest polymeric stress. This is because the axisymmetric configuration has the strongest contraction in terms of the ratio of pore throat velocity to upstream velocity.

However, no upstream recirculation is observed in the 3D channel with strong confinement, consistent with the findings reported by Oliveira et al. (2008). This may arise from the additional viscous stresses generated by the confining walls in the 3D geometry, which stabilize perturbations to the streamlines. The flow in the 3D configuration is always strongly confined by the top and bottom walls, whereas the other two cases are much less confined away from the contraction. As a result, there is least change in confinement in the 3D case. In this study, the confinement effect in the 3D channel is also investigated for various aspect ratios (AR=$H_c/L_c$) at the same value of $\textrm{Wi}~(=24)$ in Appendix B. A weaker confinement shifts the behavior in 3D toward that of the 2D case (as AR$\rightarrow \infty$). Compared to the 2D case, vortices are not significantly suppressed in the axisymmetric configuration, instead showing a larger recirculation zone at the same $\textrm{Wi}$, as illustrated in Fig. 9a and b.

#### Flow Topology Parameter

Figure 8d–f illustrates the spatial distributions of the flow topology parameter *Q* (as defined in Eq. (9)) in the Carreau fluid. For the axisymmetric and 2D cases, elongational flows ($Q\rightarrow 1$) are distributed along the centerline, while simple shear flow ($Q=0$) is observed near the walls. In contrast, the elongational flow regime becomes wider upstream and downstream due to the limited depth in the 3D case. The confined geometry of the 3D channel enhances the presence of elongational flow ($Q\rightarrow 1$) at the mid-depth plane within the region of interest (defined in Fig. 7a).

**Fig. 10:**
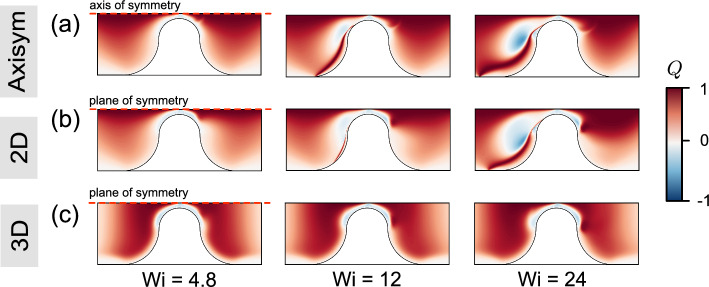
Spatial distribution of the flow topology parameter *Q*, Eq. (9), in **a** an axisymmetric channel; **b** 2D channel; **c** 3D channel (mid-depth plane) at different values of $\textrm{Wi}$ for rheology “FENE-P 1”. $\textrm{Wi}=\lambda _\textrm{F}U_c/L_c$, where $U_c$ is the interstitial velocity, defined as $U_c=U_\textrm{in}(A_\textrm{channel}/A_\textrm{const})$. In axisymmetric and 2D geometries, upstream flow becomes more complex represented by flow topology parameter *Q*. Elongational flow appears to dominate, particularly in the upstream region, suggesting that extra shear stresses are produced, which in turn inhibit vortex formation

**Fig. 11:**
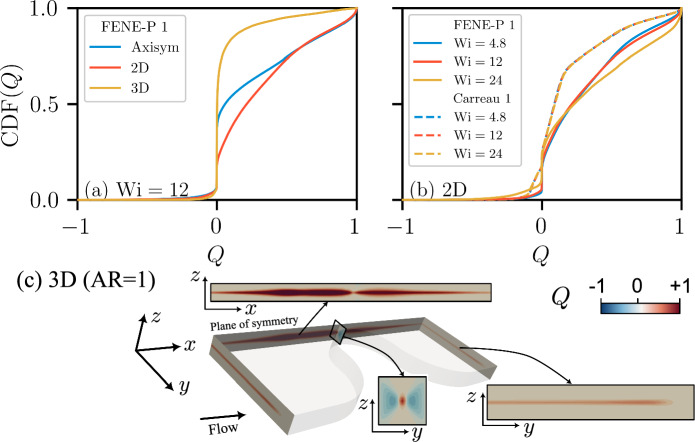
**a** Cumulative distribution function (CDF) of the flow topology parameter *Q* within the whole computational domain for flow geometries for rheology “FENE-P 1”. For the 3D geometry, simple shear flow appears to be dominant, indicating more shear stresses induced by the confining walls. **b** CDF(*Q*) within the whole computational domain for the 2D geometry for rheologies “FENE-P 1” and “Carreau 1” at three different values of $\textrm{Wi}$. The global distribution of *Q* evolves with $\textrm{Wi}$ for the FENE-P rheology, whereas it remains essentially unchanged for the Carreau fluid. **c** Spatial distribution of *Q* in the 3D geometry with AR = 1 for rheology “FENE-P 1” at $\textrm{Wi}=24$. In the 3D case, the flow becomes less extensional and more dominated by shear nearer the top and bottom walls

To link the recirculation formation that occurs for the FENE-P fluid (Sect. 3.1.1) to the flow topology, Figure 10 presents the spatial distribution of the flow topology parameter *Q* for the same scenarios as in Fig. 9. The results show that viscoelastic flow appears to be characterized by the strong presence of simple shear flow ($Q=0$) and elongational flow ($Q=+1$). Compared with Fig. 8d–f, the spatial distributions of *Q* differ from those of the Carreau fluid at the same value of $\textrm{Wi}~(=24)$, especially within the upstream regime. For $\textrm{Wi}=24$, rotational flows ($Q\rightarrow -1$) feature prominently in the axisymmetric and 2D cases, as does a band of strong elongational flow along the upstream face of the constriction. The development of upstream rotational and elongational flows can be closely linked to the development of upstream recirculation (see Fig. 9). For the 3D channel, the *Q* field for the FENE-P fluid is similar to that for the Carreau fluid. Away from the mid-plane (i.e., nearer the top and bottom walls), the flow becomes significantly less extensional and more dominated by shear (see Fig. 11c), in agreement with the findings of Zografos et al. (2020). This suggests that the confining walls promote the dominance of shear stresses, which in turn is associated with the suppression of vortices.

To quantitatively compare the global flow topology across different rheologies and flow geometries, we next consider the associated cumulative distribution function CDF(*Q*) for the entire computational domain (see Fig. 7a). Figure 11a demonstrates that the flow geometry can significantly change the distribution of *Q*. In the 3D channel, the simple shear flows are centrally distributed ($Q\rightarrow 0$). In 2D and axisymmetric geometries, in contrast, a higher proportion of flow is between the simple shear and pure elongational flow regimes ($Q\in [0,1]$). Figure 11b considers the flow rheology and the value of $\textrm{Wi}$ for the 2D geometry. The CDF(*Q*) for the Carreau rheologies overlap for all values of $\textrm{Wi}$, while the FENE-P rheologies evolve from simple shear ($Q=0$) toward increasingly rotational/elongational flows ($Q\rightarrow \pm 1$) (but not reaching, $Q=-1$). This indicates that more complex and mixed flow topologies are expected with increasing elastic effects (varying $\textrm{Wi}$).

#### Polymer Fluid Conductance and Recirculation Zone

**Fig. 12:**
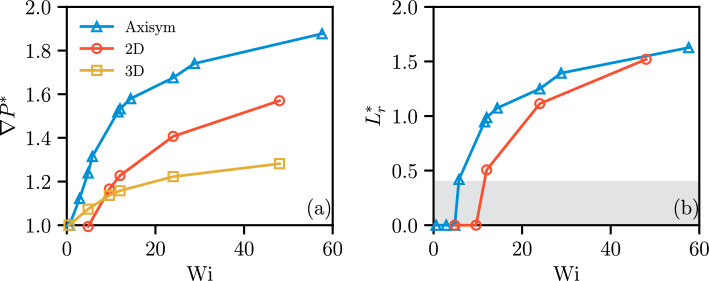
**a** Relative pressure gradient versus $\textrm{Wi}$ for rheology “FENE-P 1” across the three flow geometries ($\nabla P^*=\nabla P_\mathrm {FENE-P}/\nabla P_\textrm{Carreau}$). $\nabla P^*$ increases monotonically as $\textrm{Wi}$ increases. For a fixed value of $\textrm{Wi}$, the axisymmetric geometry produces the highest $\nabla P^*$, while the 3D geometry yields the lowest. **b** Evolution of relative recirculation length ratio $L_r^*$ against $\textrm{Wi}$ for rheology “FENE-P 1” in the two flow geometries where recirculation occurs: axisymmetric and 2D geometries ($L_r^*=L_r/(W/2)$). Both geometries exhibit similar trends; however, $L_r^*$ increases more steeply with $\textrm{Wi}$ in the 2D geometry than in the axisymmetric geometry. Note that $L_r^*\le 0.4$ (shaded region) corresponds to no recirculation

Figure 12a compares the responses for the rheologies “Carreau 1” and “FENE-P 1” by considering the relationship between the relative pressure gradient $\nabla P^*$ and $\textrm{Wi}$ for the three flow geometries ($\nabla P^*$ is the ratio of the pressure gradient in the FENE-P rheology to that in the Carreau rheology). The value of $\nabla P^*$ is close to unity for small $\textrm{Wi}$, suggesting that the two rheologies behave similarly when viscoelastic effects are weak. The relative pressure gradient $\nabla P^*$ then increases strongly and monotonically as $\textrm{Wi}$ increases, indicating that the viscoelastic FENE-P rheology is associated with a decreasing fluid conductance and an increasing energy dissipation than the purely shear-thinning Carrreau rheology as $\textrm{Wi}$ increases. For moderate to large $\textrm{Wi}$, the value of $\nabla P^*$ is largest for the axisymmetric case and smallest for the 3D case, reaching maximum values at $\textrm{Wi}=48$ of around 1.25, 1.6 and 1.8 for the 3D, 2D, and axisymmetric geometries, respectively. Varying the level of confinement alters the $\nabla P^*$ response, transitioning the system from a 3D to a 2D regime at a given value of $\textrm{Wi}$, as detailed in Appendix B. This may account for the differences observed between the 2D and 3D data in Fig. 12a, which arise from the effect of confinement.

The evolutions of the recirculation zone ($L_r^*=L_r/(W/2)$) against $\textrm{Wi}$ for the axisymmetric and 2D cases are illustrated in Fig. 12b. Both configurations display comparable evolution patterns; nevertheless, for a fixed $\textrm{Wi}$, the axisymmetric case produces a larger recirculation region than the 2D configuration. For both geometries, the rate of increase decreases as $\textrm{Wi}$ grows, and the flow may approach a steady recirculation region. For the same value of $\textrm{Wi}$, upstream recirculation is more prone to occur in the axisymmetric configuration than in the 2D case.

### Effects of $\beta ^{-1}$

**Fig. 13:**
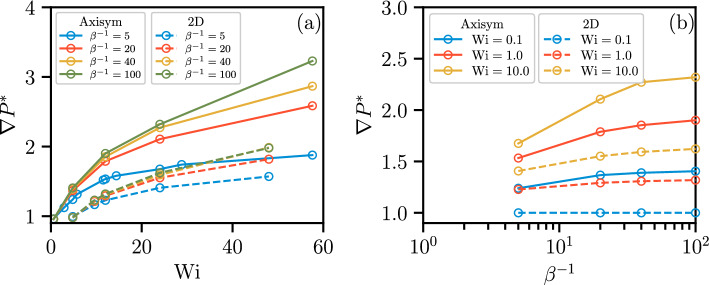
**a** Relative pressure gradient $\nabla P^*$ against $\textrm{Wi}$ for the four different values of $\beta ^{-1}$ for 2D and axisymmetric geometries. **b** $\nabla P^*$ against $\beta ^{-1}$ for different values of $\textrm{Wi}$ for 2D and axisymmetric geometries. $\nabla P^*$ increases as $\beta ^{-1}$ grows, and approaches a plateau for large values of $\beta ^{-1}$. The axisymmetric case (solid line) shows a more pronounced sensitivity to variations in $\beta ^{-1}$

**Fig. 14:**
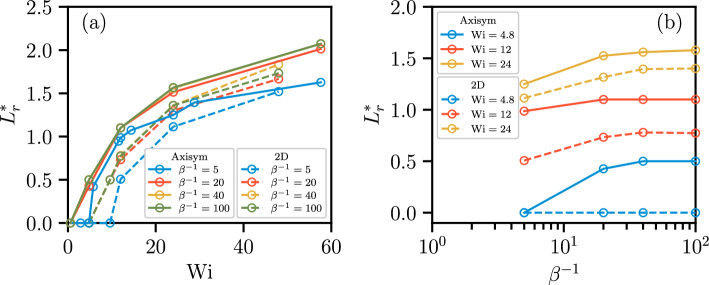
**a** Recirculation length ratio $L_r^*$ against $\textrm{Wi}$ for the four values of $\beta ^{-1}$ for axisymmetric and 2D geometries. $L_r^*$ grows significantly and monotonically as $\textrm{Wi}$ increases. For a moderate value of $\textrm{Wi}$, the axisymmetric configuration exhibits a larger $L_r^*$ than the 2D configuration. **b** $L_r^*$ against $\beta ^{-1}$ for different values of $\textrm{Wi}$ for axisymmetric and 2D geometries. $L_r^*$ grows as $\beta ^{-1}$ increases and eventually saturates at a constant value, indicating a slower rate of vortex formation

The shear-thinning strength can be defined as $\beta ^{-1}=(\mu _\textrm{s}+\mu _\textrm{p})/\mu _\textrm{s}=\mu _0/\mu _\infty$ for rheologies FENE-P and Carreau, respectively. As listed in Table 1, four values of $\beta ^{-1}$ are considered to explore the effects of shear-thinning strength on polymer fluid flow. Figure 13(a) presents the relationship between $\nabla P^*$ and $\textrm{Wi}$ for different $\beta ^{-1}$ values in the axisymmetric geometry. As the polymeric contribution to the zero-shear-rate viscosity ($\mu _0$) increases (that is, $\beta ^{-1}$ increases), $\nabla P^*$ increases at a given Weissenberg number. At a given value of $\textrm{Wi}$, $\nabla P^*$ increases with increasing shear-thinning strength $\beta ^{-1}$, approaching a plateau value at large $\beta ^{-1}$ (Fig. 13b). This observation indicates that, as $\beta ^{-1}$ increases, the growth of the pressure gradient across porous media predicted by the FENE-P rheology becomes less pronounced than that predicted by the Carreau rheology. As depicted in Fig. 14a, the relative recirculation length, $L_r^*$, increases as $\beta ^{-1}$ increases. At the same $\beta ^{-1}$ value, the axisymmetric geometry exhibits a larger value of $L_r^*$ compared to the 2D geometry. As $\beta ^{-1}$ increases, $L_r^*$ shows a trend similar to $\nabla P^*$, increasing toward a plateau value at large $\beta ^{-1}$ (Fig. 14b). The results indicate that the response of polymer fluid conductance and the recirculation zone induced by the practically used polymer solutions can be anticipated and related to real-world conditions by aligning the relevant non-dimensional groups.

**Fig. 15:**
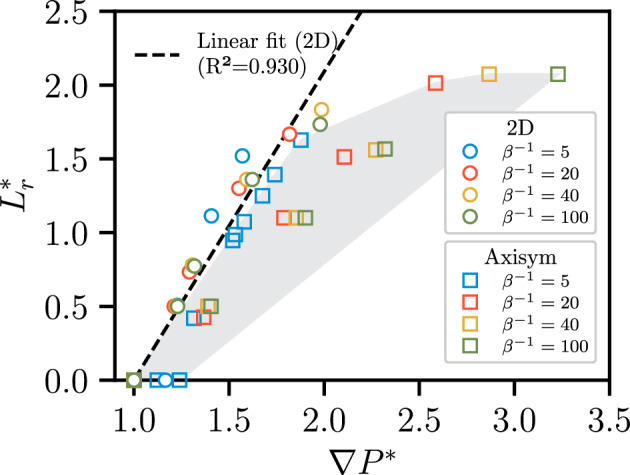
Correlation between $L_r^*$ and $\nabla P^*$ for the results presented in Figs. 13 and 14 for both axisymmetric and 2D geometries. $L_r^*$ increases monotonically with $\nabla P^*$. The shaded area indicates the scattering region for the axisymmetric geometry, which exhibits high variability, whereas the 2D geometry displays a clear linear correlation. For the same recirculation zone, the axisymmetric geometry exhibits greater dissipation than the 2D geometry

To better understand the correlation between energy dissipation and the development of recirculation, $L_r^*$ is plotted against $\nabla P^*$ in Fig. 15. The quantity $L_r^*$ is roughly proportional to $\nabla P^*$. The relative recirculation length $L_r^*$ increases more steeply with $\nabla P^*$ for the 2D geometry than for the axisymmetric geometry, indicating that the recirculation zone is larger in the 2D case than in the axisymmetric case for a given $\nabla P^*$. In this context, a recirculation zone of the same size is more dissipative in the axisymmetric configuration over the 2D geometry.

## Packed Sphere System

**Fig. 16:**
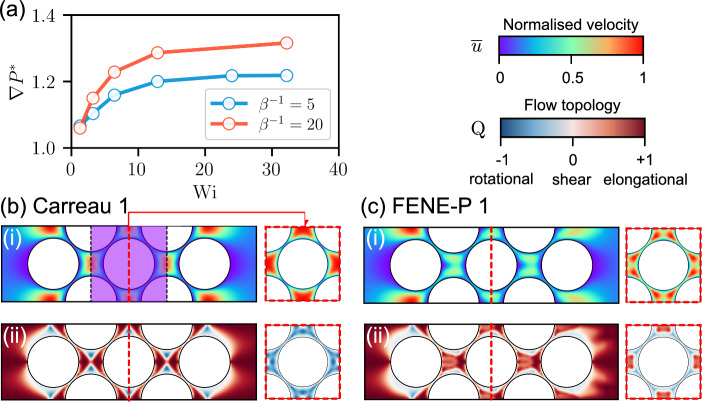
**a** Relationship between $\nabla P^*$ and $\textrm{Wi}$ for $\beta ^{-1}=5$ and 20. $\textrm{Wi}=\lambda _\textrm{F}U_c/d_s$, where $U_c$ is the interstitial velocity, $U_c=U_\textrm{in}/\phi$, and $d_s$ is the distance between adjacent particles. Distribution of (i) normalized velocity magnitude $\overline{u}=\vert u|/\vert u|_\textrm{max}$ and (ii) flow topology parameter *Q* in a **b** Carreau fluid; and **c** FENE-P fluid ($\beta ^{-1}=5$) on two orthogonal planes in an FCC packing at $\textrm{Wi}=24$ ($U_\textrm{in}\approx 3.7\times 10^{-4}~\mathrm {m/s}$). The red dashed line indicates the chosen square cross section. For the Carreau fluid, the flow is expected to follow regular patterns, whereas the FENE-P fluid shows more intricate flow behavior both overall and within individual pores

We next perform simulations of a fully 3D, face-centered cubic (FCC) packing of 23 uniform spheres, using periodic boundaries to mimic a system of infinite lateral extent (Fig. 3a). A region of interest was selected to determine the pressure gradient across the packing, as highlighted in Fig. 16b. Figure 16a illustrates the relationship between the relative pressure gradient and $\textrm{Wi}$ for the FCC packing for $\beta ^{-1}=5$ and 20 (Table 1). The responses in the FCC packing exhibit a trend similar to the 3D single-constriction channel (Fig. 12a). In particular, for a given value of $\textrm{Wi}$, the relative pressure gradient ($\nabla P^*$) in an FCC packing is comparable in magnitude with that in a 3D constriction channel (compare Figs. 12a and 16a). Comparing the results for $\beta ^{-1}=5$ with those for $\beta ^{-1}=20$ suggests that $\nabla P^*$ increases with $\beta ^{-1}$ in an FCC packing, as also found above for a single constriction (see Figs. 13 and 14).

No flow recirculation was observed within the pore space, most likely because of the narrow gap between adjacent spheres. Within individual pores, the enclosing spheres induce higher shear stresses, which inhibit vortex formation (the suppression mechanism resembles that observed in the 3D geometry in Sect. 3). In Fig. 16b and c, we present the distribution of the normalized velocity and the flow topology parameter *Q* on two orthogonal cross sections for the Carreau and FENE-P fluids, respectively. The Carreau fluid exhibits a regular distribution of $\bar{u}$ in each pore, while the FENE-P fluid exhibits a more complex distribution of $\bar{u}$ with $\bar{u}$ being larger upstream of a sphere than at the same distance downstream. In the Carreau fluid, the maximum $\bar{u}$ occurs in the pore center, whereas in a FENE-P fluid, there are two local maxima. This feature may be due to the stretching of polymer chains in the upstream pore spaces and in the wake of the sphere, partially blocking the pore space. The distribution of *Q* in the Carreau simulation and the corresponding FENE-P simulation are illustrated in Fig. 16b(ii) and c(ii), respectively. In an individual pore, the results show a regular distribution of *Q* in the Carreau fluid, while more complex flow topologies are induced in the FENE-P fluid. Elongational flows are distributed more widely in the FENE-P fluid compared to the Carreau fluid, indicating that more energy is dissipated in the pore space because of the more complex flow topology.

## Conclusions

This study has considered the effects of viscoelasticity on polymer fluid flow in porous media using direct, pore-scale CFD simulations. The flow of a viscoelastic FENE-P model was compared with a corresponding shear-thinning Carreau model in three geometries, 2D, quasi-3D and axisymmetric, as well as in a 3D FCC packing of spheres. The key conclusions are as follows:

- In terms of polymer fluid conductance, a higher pressure drop is required for the FENE-P fluid to achieve the same flow velocity, in comparison with its corresponding Carreau fluid. This suggests that viscoelasticity leads to a decrease in polymer fluid conductance. Furthermore, the axisymmetric geometry exhibits the largest drop in polymer fluid conductance compared to the 2D and 3D cases.
- Geometry controls the flow pattern of a viscoelastic fluid. 3D geometries with strong confinement (nearby walls) suppress recirculation and vortex formation at low $\textrm{Re}$. In contrast, axisymmetric and 2D cases result in upstream recirculation, since the geometries are less restrictive. Greater polymer chain stretching hinders polymer fluid flow across the constriction center, leading to recirculation.
- Increasing shear-thinning strength $\beta ^{-1}$ can amplify the pressure gradient in the FENE-P fluid relative to the corresponding Carreau fluid without viscoelasticity. Beyond a critical shear-thinning strength, less pronounced upstream recirculation is developed.
- The relative recirculation length $L_r^*$ varies approximately linearly with the relative pressure gradient, with $L_r^*$ increasing more rapidly with $\nabla P^*$ in the 2D geometry than in the axisymmetric geometry.
- In a regular (FCC) packing of spheres, there are excess energy dissipation and asymmetric flow patterns both upstream and downstream, and within individual pores. This suggests that polymer chain stretching and elongation can partially obstruct the 3D pore space, resulting in more intricate flow characteristics than those seen with inelastic shear-thinning fluids.

Our results suggest that microfluidic tests in thin, quasi-2D (i.e., strongly confined) flow geometries may not be adequate to quantify viscoelastic effects in realistic 3D porous media. Viscoelasticity may partly account for the additional energy dissipation, helping to explain the previously observed mismatch between the effective viscosity obtained from shear rheology and that inferred from flow experiments in porous media (Ejezie et al. 2021; Wang et al. 2025). The data suggest that the combined effect of elastic and shear-thinning may provide more detailed insight into the use of polymer fluids in ground engineering.

## Figures and Tables

**Fig. 17:**
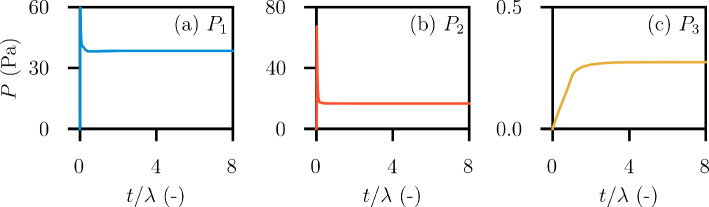
**a**–**c** Variation in pressure against time ($t/\lambda _\textrm{F}$) at three different positions in an axisymmetric case at $\textrm{Wi}=24$ for rheology “FENE-P 1”. Stable flow conditions are maintained throughout the simulation period, indicating that time-dependent effects can be disregarded

**Fig. 18:**
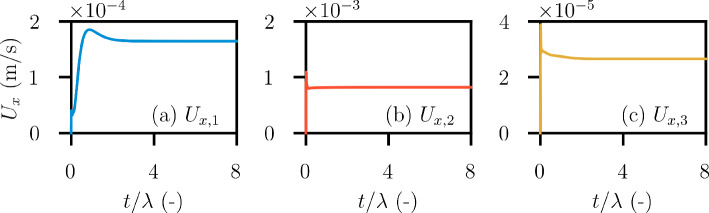
**a**–**c** Variation in *x*-direction velocity component against time ($t/\lambda _\textrm{F}$) at three different positions in an axisymmetric case at $\textrm{Wi}=24$ for rheology “FENE-P 1”. Stable flow conditions are maintained throughout the simulation period, indicating that time-dependent effects can be disregarded

**Fig. 19:**
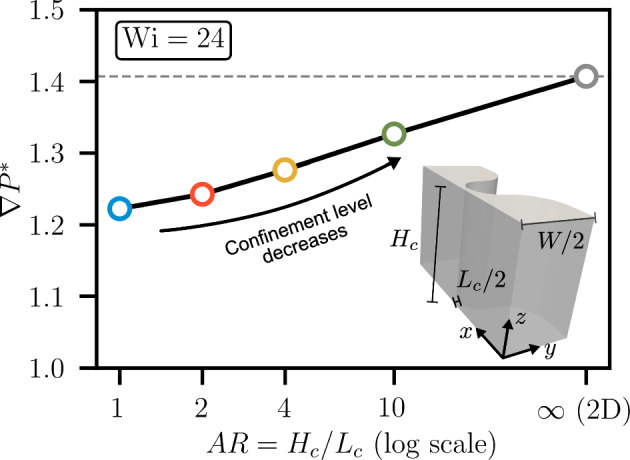
Evolution of the relative pressure gradient $\nabla P^*$ $(=\nabla P_\mathrm {FENE-P}/\nabla P_\textrm{Carreau})$ as the confinement level changes in a 3D example at $\textrm{Wi}=24$. $\nabla P^*$ increases as AR becomes larger (reduced confining effect), tending toward the value corresponding to the 2D case. This progression can account for the discrepancies between the 2D and 3D cases shown in Fig. 12a

**Fig. 20:**
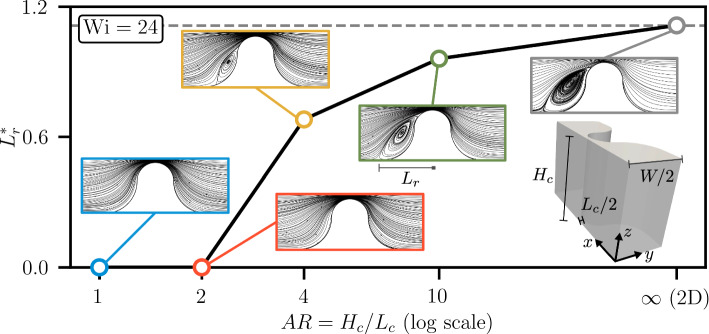
Evolution of the relative upstream recirculation length, $L_r^* = L_r/(W/2)$), with varying confinement levels in the 3D configuration at $\textrm{Wi}=24$. At low AR values (AR = 1 and 2), no upstream recirculation is observed, due to the strong confinement imposed by the top and bottom walls. Once the aspect ratio AR exceeds a critical value (within the range AR = [2, 4]), upstream recirculation regions appear and grow as the confining effects weaken (i.e., for higher AR), tending toward the behavior observed in the 2D configuration

## Data Availability

The viscoelastic solver RheoTool used in this study can be accessed through https://github.com/fppimenta/rheoTool. Representative input files for OpenFOAM simulations are available from Zenodo (https://zenodo.org/). The DOI link is 10.5281/zenodo.17653445.

## Temporal Effects

In this study, a conservative value of $L^2=10$ (see Table 1) has been used to avoid significant temporal effects that would be seen in a viscoelastic fluid using a larger $L^2$ (Kumar et al. 2021). Figure 17a–c illustrates the variation in pressure with time in three monitor probes: (1) upstream; (2) in the constriction center; and (3) downstream. The relationship between velocity and non-dimensional time ($t/\lambda$) is presented in Fig. 18a–c. The simulation data verify an effective convergence analysis with the adopted FENE-P parameters, without noticeable temporal effects.

## Confinement in 3D

To explore how the viscoelastic flow dynamics evolves with the height of the channel in the 3D example (Fig. 2b), we systematically vary the channel height and analyze the resulting instabilities (relative pressure gradient $\nabla P^*$ and relative recirculation length $L^*_r$). The aspect ratio (AR) between the channel height and the constriction size is given by AR = $H_c/L_c$. Five values of AR are considered to assess the effect of the confinement level, AR = $[1,2,4,10,\infty ]$. When AR = 1, the geometric arrangement corresponds to the three-dimensional case examined in this work; as AR $\rightarrow \infty$, the configuration gradually changes from a 3D to a 2D case.

Figure 19 presents the variation in the relative pressure gradient, $\nabla P^*$ against the values of AR at $\textrm{Wi}=24$. The value of $\nabla P^*$ at AR = 1 represents the three-dimensional configuration shown, whereas AR = $\infty$ denotes the two-dimensional case illustrated in Fig. 12a. As AR increases, reflecting a weaker confining effect, the value of $\nabla P^*$ rises and tends toward the characteristics of a 2D case. The evolution of upstream recirculation with different confinement levels is illustrated in Fig. 20. There exists a critical value of AR (located within the interval AR = [2, 4]), above which upstream recirculation occurs. For larger values of AR (where the confining effect is less significant), the recirculation zone expands and tends toward the behavior observed in the 2D case.

## List of symbols

AR: Ratio between the height of the single-constriction channel and the constriction size ($=H_c/L_c$)
$$\mu _a$$: Apparent viscosity ($$\mathrm {Pa~s}$$)
$\mu _\infty$: Infinite-shear-rate viscosity ($\mathrm {Pa~s}$)
$\mu _0$: Zero-shear-rate viscosity ($\mathrm {Pa~s}$)
$$\dot{\gamma }$$: Shear rate ($$\mathrm {s^{-1}}$$)
*n*: The power-law exponent in the Carreau model
$\lambda$: Time constant in the Carreau model ($\textrm{s}$)
$\tau _\textrm{p}$: Polymeric extra-stress tensor
$\mu _\textrm{p}$: Polymeric contribution to zero-shear rate viscosity ($\mathrm {Pa~s}$)
$$\mu _\textrm{s}$$: Solvent viscosity ($$\mathrm {Pa~s}$$)
$\lambda _\textrm{F}$: Relaxation time of the polymer in the FENE-P rheology model ($\textrm{s}$)
*I*: Identity tensor
$$\frac{D}{Dt}$$: Material derivative
$\nabla$: Upper-convected time derivative
*f*: Function in the FENE-P rheology model
$L^2$: Measurement of extensibility of polymer chains
$R_0$: Maximum length of the polymer chain ($\textrm{m}$)
$R_\textrm{e}$: Equilibrium length of the polymer chain ($\textrm{m}$)
$$\beta $$: Viscosity ratio
$\beta ^{-1}$: Strength of shear thinning of the fluid
$$\rho $$: Fluid density ($$\mathrm {kg/m^3}$$)
$\textrm{u}$: Fluid velocity vector
*p*: Fluid pressure ($$\textrm{Pa}$$)
$\tau$: Deviatoric stress tensor in Eq. 2 ($\textrm{Pa}$)
$\nabla \boldsymbol{\textrm{u}}$: Velocity gradient tensor
$\textrm{D}$: Rate-of-strain tensor
*t*: Time ($$\textrm{s}$$)
$\tau _\textrm{p}$: Polymeric contribution to the extra-stress tensor $\boldsymbol{\tau }$ ($\textrm{Pa}$)
$\tau _\textrm{s}$: Solvent contribution to the extra-stress tensor $\boldsymbol{\tau }$ ($\textrm{Pa}$)
*D*: Diameter of a capillary tube ($\textrm{m}$)
$$\textrm{Re}$$: Reynolds number
$U_\textrm{in}$: Inlet flow velocity ($\mathrm {m/s}$)
$$L_\textrm{c}$$: Constriction size ($$\textrm{m}$$)
*W*: Width of single-constriction channel ($\textrm{m}$)
*L*: Total length of single-constriction channel ($\textrm{m}$)
$H_c$: Height (or thickness) of single-constriction channel ($\textrm{m}$)
$$\textrm{Wi}$$: Weissenberg number
$U_c$: Flow velocity within the constriction (interstitial velocity) ($\mathrm {m/s}$)
$A_\textrm{channel}$: Cross-sectional area of single-constriction channel ($\mathrm {m^2}$)
$A_\textrm{const}$: Cross-sectional area of constriction ($\mathrm {m^2}$)
$\phi$: Porosity of the sphere packing
$d_s$: Distance between adjacent particles ($\textrm{m}$)
$$\nabla P$$: Pressure gradient, $$=\Delta P/\Delta L$$ ($$\mathrm {Pa/m}$$)
$\nabla P_\mathrm {FENE-P}$: Pressure gradient in the FENE-P fluid ($\mathrm {Pa/m}$)
$\nabla P_\textrm{Carreau}$: Pressure gradient in the Carreau fluid ($\mathrm {Pa/m}$)
$\nabla P^*$: Relative pressure gradient, $=\nabla P_\mathrm {FENE-P}/\nabla P_\textrm{Carreau}$
$L_{sc}$: Channel length considered for the calculation of the pressure drop ($\textrm{m}$)
$L_r$: Horizontal distance from the left edge of upstream recirculation to the center of constriction ($\textrm{m}$)
$L_r^*$: Relative recirculation length, $=L_r/(W/2)$
*Q*: Flow topology parameter
$\boldsymbol{\textrm{W}}$: Rate of rotation tensor
$U_\textrm{max}$: Maximum flow velocity ($\mathrm {m/s}$)
$\overline{U}$: Average flow velocity (inlet flow velocity) ($\mathrm {m/s}$)
$$\bar{u}$$: Normalized velocity, $$=\vert u|/\vert u|_\textrm{max}$$
$\textrm{tr}(\boldsymbol{\tau }_\textrm{p})$: Trace of the polymeric stress tensor ($\textrm{Pa}$)
$\tau _{xx}$: *x*-direction normal stress ($\textrm{Pa}$)
$\tau _{yy}$: *y*-direction normal stress ($\textrm{Pa}$)
$\tau _{zz}$: *z*-direction normal stress ($\textrm{Pa}$)
*K*: Permeability of the packed sphere system ($\textrm{m}^2$)
*R*: Particle radius ($$\textrm{m}$$)
$$\Delta d$$: Mesh size ($$\textrm{m}$$)
